# The ability of the Knee Osteoarthritis Outcome Score to detect changes over time is limited in patients with patellar instability due to substantial ceiling effect

**DOI:** 10.1002/jeo2.70146

**Published:** 2025-04-01

**Authors:** Trine Hysing‐Dahl, Anne Gro Heyn Faleide, Per Arne Skarstein Waaler, Eivind Inderhaug

**Affiliations:** ^1^ Department of Surgery Haraldsplass Deaconess Hospital Bergen Norway; ^2^ Department of Clinical Medicine University of Bergen Bergen Norway; ^3^ Orthopedic Clinic Haukeland University Hospital Bergen Norway

**Keywords:** COSMIN, Norwich Patellar Instability score, patellar instability, reliability, validity

## Abstract

**Purpose:**

The purpose of the current study was to evaluate important aspects of interpretability (floor and ceiling effects) for the Knee injury and Osteoarthritis Outcome Score (KOOS) in patients with patellar instability. Secondarily, the study aims to provide minimal important clinical difference (MICD) values for all subscales in this patient category.

**Methods:**

Patients undergoing patella stabilising surgery with an individualised approach based on anatomic deviation were prospectively included if (1) ≥13 years of age at the time of surgery, (2) fluent in Norwegian and (3) able to understand and complete the questionnaires. Patients were excluded if they had concomitant bony and/or knee ligament injuries. KOOS was completed before and 6 months after surgery. *Interpretability* of the KOOS was evaluated according to recommendations from COnsensus‐based Standards for the selection of health Measurement INstruments. A *floor or ceiling effect* is considered to be present if the number of patients that had a score in the lower (0–10) or upper (90–100) end of the scale exceeded 15%. This was identified with a distribution‐based approach with standard deviation (SD) of the change score between pre‐ and postoperative scores using the following equation: MICD = 0.5 × SD.

**Results:**

A substantial ceiling effect was present in the KOOS subscales *pain* and activities of daily living (*ADL*) measured prior to surgery, and in all, except the quality of life subscale, 6 months after surgery. KOOS *ADL* demonstrated the highest number of patients, 46% preoperatively and 72% postoperatively with a ceiling effect. In addition, 32% of patients had the best possible score on the *pain* subscale 6 months after surgery. The only subscale that displayed a floor effect was the preoperative KOOS *Sport/Rec*. The MICD for the different subscales ranged from 7.6 to 12.4.

**Conclusion:**

The substantial ceiling effect in the current implies that the KOOS is not suited to evaluate the long‐term effect of treatment in patients with patellar instability.

**Level of Evidence:**

Level II.

**Abbreviations**
ACL: anterior cruciate ligament
BPII: Banff Patellofemoral Instability Instrument
COSMIN: COnsensus‐based Standards for the selection of health Measurement INstruments
KOOS: Knee injury and Osteoarthritis Outcome Score
KOOS‐PF: patellofemoral pain and osteoarthritis subscale
MICD: minimal important clinical difference
MPFL‐R: medial patellofemoral ligament reconstruction
NPI: Norwich Patellar Instability
PASS: patient acceptable symptom state
PROM: patient‐reported outcome measures
QOL: quality of life
SCB: substantial clinical benefit
SD: standard deviation

## INTRODUCTION

Lateral patellar dislocation is a disabling disorder, mostly affecting adolescents and young adults with an incidence of 42 per 100,000 [7]. Despite increasing research interest in this group of patients, clinical outcome evaluation remains relatively inconsistent. Patient‐reported outcome measures (PROMs) used to evaluate the effectiveness of treatments and patient recovery in clinical practice and research do not adequately address the needs of this group [9]. In the absence of diagnose‐specific PROMs for these patients, the Knee injury and Osteoarthritis Outcome Score (KOOS) has been used for decades to analyse the postoperative outcomes in patients with patellar instability [2, 4, 12, 13, 14, 18, 19, 20, 28]. This knee‐specific questionnaire was initially developed to evaluate longitudinal changes in knee function and impairments related to early onset of osteoarthritis in patients with anterior cruciate ligament (ACL) tear [24]. The 42 items of KOOS represent a significant responder burden for patients [30]. Consequently, several shorter versions exist, for example, the patellofemoral pain and osteoarthritis subscale (KOOS‐PF) and KOOS Global [5, 11]. Despite its widespread use, information regarding the psychometric properties of KOOS in patients with patellar instability remains limited.

Today's increasing knowledge in clinimetrics and the emergence of new, validated and diagnoses‐specific PROMs for patients with patellar instability—such as the Banff Patellofemoral Instability Instrument (BPII) [8] and the Norwich Patellar Instability (NPI) score [9, 27]—calls for a careful assessment of which PROMs that are best suited for evaluating patients with this condition. To secure the high quality of the instruments we use in clinical practise and research, the COnsensus‐based Standards for the selection of health Measurement INstruments (COSMIN) initiative have outlined criteria for evaluating the quality of a questionnaire's psychometric properties [17]. One important aspect of such evaluation is to evaluate a PROM's interpretability.

The term interpretability refers to ‘the degree to which one can assign qualitative meaning—that is, clinical or commonly understood connotations—to an instrument's quantitative scores or changes in scores’ [6, p. 228] indicating the scores or change scores meaning is clear. Information on how we should interpret a PROM score includes floor and ceiling effects, the minimal important clinical difference (MICD), substantial clinical benefit (SCB) and patient acceptable symptom state (PASS). Even though a substantial ceiling effect was identified in one study [32] and MICD, SCB and PASS values for KOOS have been suggested for patients undergoing medial patellofemoral ligament reconstruction and tibial tubercle osteotomies [23, 33], information on interpretability in patients with patellar instability is still scarce. One example is MICD values needed in patients treated with an individualised approach, in addition to examination of floor and/or ceiling effects in more populations. Further validation of these aspects is necessary to determine whether the KOOS is an appropriate instrument for this patient group.

The purpose of the current study was therefore to evaluate a crucial aspect of interpretability for the KOOS in patients with patellar instability: floor and ceiling effects. Secondarily, the study aims to provide MICD values for all KOOS subscales in this patient category.

## MATERIALS AND METHODS

Patients undergoing patella stabilising surgery were prospectively included from the orthopaedic unit in three hospitals in Norway. Inclusion criteria included: (1) ≥13 years of age at the time of surgery, (2) fluent in Norwegian and (3) able to understand and complete the questionnaires. They were excluded if they had concomitant bony and/or knee ligament injuries. Patients completed the KOOS before and 6 months after patellar stabilising surgery.

### The KOOS

The original KOOS comprises 42 questions across five subscales, where each item is scored on a five‐point Likert scale and reported separately: pain (nine items), symptoms (seven items), activities of daily living (ADL) (17 items), sport and recreation function (five items) and knee‐related quality of life (QOL) (four items). A sum score is made for each subscale where higher scores reflect better function [24].

### Surgical procedures

An individualised surgical approach (a la carte) based on anatomic deviation was used. The type of surgery was based on findings from the preoperative counselling and radiologic examinations, including radiographs and MRI scans. All patients underwent a medial patellofemoral ligament reconstruction (MPFL‐R) by use of a gracilis autograft from the ipsilateral knee. Tibial tubercle osteotomy with distalisation or medialisation was considered in cases of patella alta or in patients with a lateralisation of the patella, measured by the tibial tuberosity‐trochlear groove distance. Finally, a thin‐flap trochleoplasty procedure was considered in cases of severe trochlear dysplasia, typically in cases with Dejour type B and D dysplasia and/or a lateral trochlear inclination of less than 11° with corresponding clinical findings.

### Ethics

The study was approved by the Regional Committee for Medical and Health Research Ethics (ID: 2020/185067), the NSD (Norwegian Centre for Research Data) Data Protection Official for Research (ID: 731409) and registered in ClinicalTrials.gov (NCT05119088). Before entering the study, all patients (or their legal guardians if <18 years) were asked to give their written informed consent prior to inclusion and data collection.

### Statistical analyses

Continuous variables are presented as mean, range and standard deviation (SD), while categorical data are presented in frequencies. Normal distribution was assessed using the Shapiro–Wilk test. The change in each subscale from pre‐ to 6 months postoperatively and differences between genders were analysed using a paired sample *t* test and an independent sample *t*‐test, respectively. The a priori significance level was set to ≤0.05, with Bonferroni corrections for multiple comparisons. Sample size determination was based on recommendations from Terwee et al. [31], suggesting a minimum of 50 patients for assessing floor or ceiling effects.

### Measurement properties

To evaluate *interpretability* of the KOOS score, recommendations from COSMIN were followed [16, 31]. A *floor or ceiling effect* is considered to be present if the number of patients with score in the lower (0–10) or upper (90–100) end of the scale exceeds 15% [15]. Each subscale of the KOOS was examined for floor and ceiling effects independently. The *MICD* refers to the minimal difference that is important and was identified with a distribution‐based approach with SD. The SD denotes the variation between groups of scores. Since a 50% SD formerly has demonstrated to be a reliable threshold for patient‐perceived change [21], the following calculation with the SD of the change score between pre‐ and postoperative scores was used; MICD = 0.5 × SD.

## RESULTS

**Figure 1:**
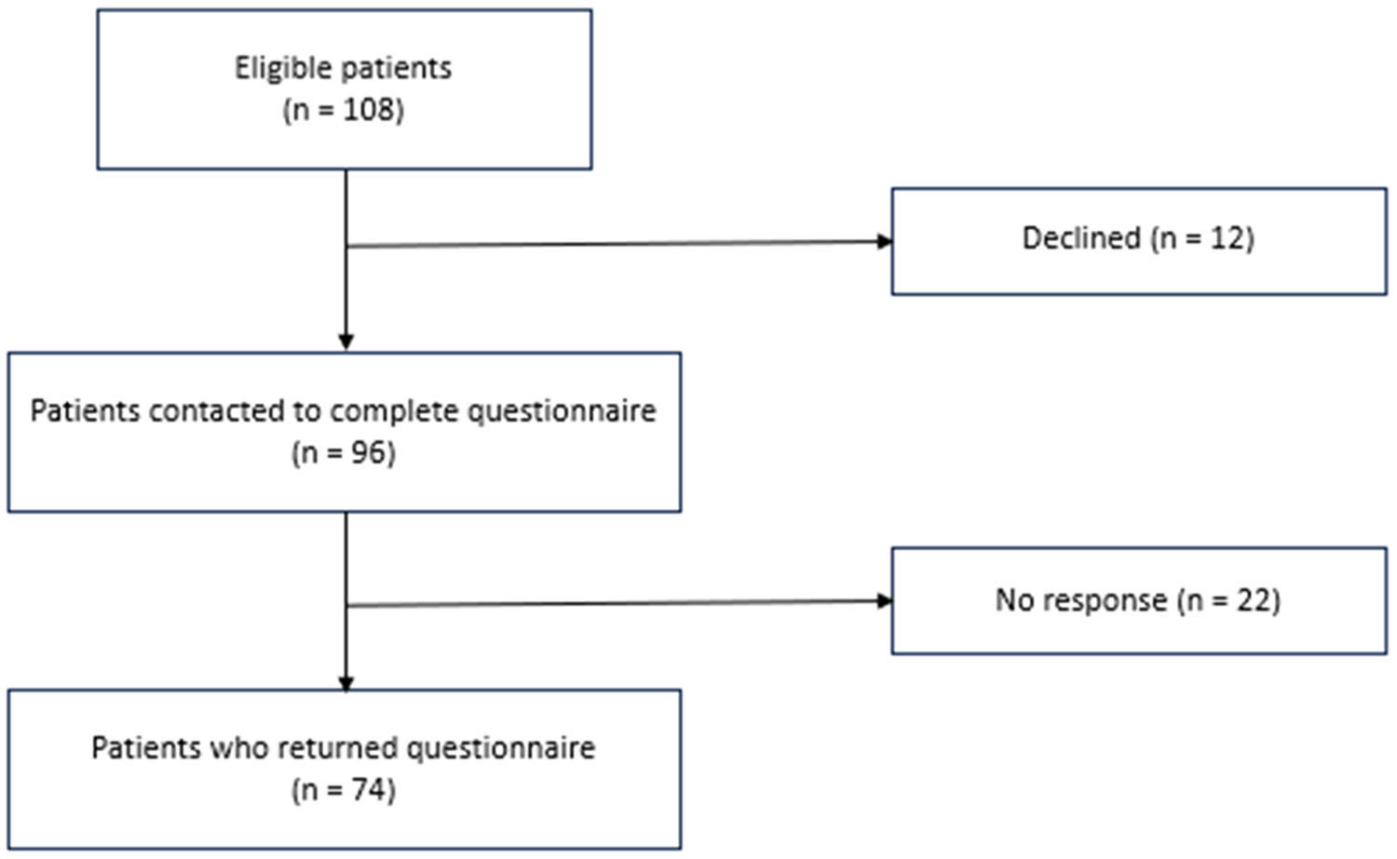
Flowchart of patient's participation.

From January 2021 to November 2023, 74 patients were included (Figure 1), and follow‐up was on average 7.3 months (±1.1) after surgery. The mean age at inclusion was 22.8 years (SD 7.6, range 13–46), 73% were female and 60% had bilateral problems (Tables 1 and 2).

**Table 1:** Descriptive statistics of patient demographics, *n* = 74.

Gender (female)	54 (73%)
Age at surgery (year)	22.8 (7.6)
Bilateral problems (yes)	44 (59.5%)
Right knee	35 (47.3%)
BMI	24.7 (5.9)
Duration of symptoms (year)	7.7 (6.8)
Surgical procedure	
MPFL‐R	9 (12%)
MPFL‐R with tuberosity tibia osteotomy	33 (45%)
MPFL‐R with trochleoplasty	17 (23%)
MPFL‐R with 2 or more concomitant procedures	14 (19%)

*Note*: Data are expressed as *n* or mean ± standard deviation unless otherwise indicated.

Abbreviations: BMI, body mass index; MPFL‐R, medial patellofemoral ligament reconstruction.

**Table 2:** Age distribution.

Age (years)	*N* (%)
<16	12 (16)
16–18	15 (20)
>18	47 (64)

The mean KOOS scores in all subscales are presented in Table 3. There was a significant improvement in all KOOS subscales from pre‐ to 6 months postoperative, all *p* < 0.001 (Table 2). The MICD for the different subscales did range from 7.6 to 12.4 (Table 3).

**Table 3:** Comparison between preoperative and postoperative KOOS scores and MICD, *n* = 74.

Subscale	Preoperative (SD)	6 months postoperative (SD)	Mean change (SD)	*p* Value^a^	MICD
KOOS pain	68.9 (21.4)	82.1 (14.7)	13.2 (19.6)	**<0.001**	9.8
KOOS symptoms	68.6 (16.1)	77.1 (13.0)	8.5 (16.5)	**<0.001**	8.2
KOOS ADL	79.4 (18.7)	91.3 (10.6)	11.9 (15.2)	**<0.001**	7.6
KOOS Sport/Rec	46.0 (27.2)	59.2 (24.6)	13.2 (24.7)	**<0.001**	12.4
KOOS QOL	32.2 (19.5)	60.4 (20.5)	28.2 (23.6)	**<0.001**	11.8

*Note*: Data are expressed as mean ± SD unless otherwise indicated. Bolded *p* value indicates a statistically significant change.

Abbreviations: ADL, activities of daily living; KOOS, Knee injury and Osteoarthritis Outcome Score; MICD, minimal important clinical difference; QOL, quality of life; SD, standard deviation.

^a^ Bonferroni adjustment for multiple comparisons.

Females had significantly more problems before surgery compared to males, except in the subscale KOOS symptoms. Six months after surgery, females still reported worse function than males, but the difference was not significant (Table 4).

**Table 4:** Comparison between gender in the five KOOS subscales pre‐ and 6 months postoperatively, *n* = 74.

Preoperative				Postoperative		
Subscale	Female (*n* = 54)	Male (*n* = 20)	*p* Value	Female (*n* = 54)	Male (*n* = 20)	*p* Value
KOOS pain	65.04 (19.8)	79.5 (22.3)	**0.009**	80.8 (14.6)	85.6 (14.6)	0.219
KOOS symptoms	66.5 (15.6)	74.4 (16.2)	0.058	76.6 (11.9)	78.5 (16.0)	0.583
KOOS ADL	76.4 (18.9)	87.6 (15.9)	**0.014**	90.4 (10.4)	93.8 (11.1)	0.235
KOOS Sport/Rec	40.4 (27.1)	61.3 (21.6)	**0.003**	57.0 (25.7)	65.3 (20.9)	0.213
KOOS QOL	28.9 (18.8)	41.3 (19.9)	**0.017**	58.2 (21.1)	66.2 (18.1)	0.139

*Note*: Data are reported as percent (*n*). Bolded *p* value indicates a significant difference between groups.

Abbreviations: ADL, activities of daily living; KOOS, Knee injury and Osteoarthritis Outcome Score; QOL, quality of life.

^a^ Bonferroni adjustment for multiple comparisons.

A substantial ceiling effect was present in the KOOS subscales *pain* and *ADL* measured prior to surgery—and in all, except the *QOL* subscale, 6 months after surgery (Table 5). KOOS *ADL* demonstrated the highest number of patients, 46% preoperatively and 72% postoperatively, scoring in the upper 10% end of the scale (ceiling effect). In addition, 32% of patients had the best possible score on the *pain* subscale 6 months after surgery. The only subscale that displayed a floor effect was the preoperative KOOS *Sport/Rec* (Table 5).

**Table 5:** Floor and ceiling effects of the five KOOS subscales pre‐ and 6 months postoperatively, *n* = 74.

Ceiling effect			Floor effect	
Subscale	Preoperative	Postoperative	Preoperative	Postoperative
KOOS pain	**11 (15%)**	**24 (32%)**	0	0
	Females 5	Females 15		
KOOS symptoms	7 (10%)	**11 (15%)**	0	0
	Females 4	Females 6		
KOOS ADL	**34 (46%)**	**53 (72%)**	0	0
	Females 21	Females 38		
KOOS Sport/Rec	6 (8%)	**11 (15%)**	**12 (16%)**	2 (3%)
	Females 3	Females 9	Females 5	Females 1
KOOS QOL	1 (1%)	7 (10%)	8 (11%)	0
	Females 1	Females 4	Females 7	

*Note*: Data are reported as the number of cases (percent). Bolded values indicates a floor or ceiling effect.

Abbreviations: ADL, activities of daily living; KOOS, Knee injury and Osteoarthritis Outcome Score; QOL, quality of life.

## DISCUSSION

The most important finding in this study was a substantial ceiling effect in most of the KOOS subscales in patients with patellar instability both before and after surgery. In addition, to provide MCID values for all five KOOS subscales in this patient group.

The extensive ceiling effect found in this study is questionable whether the KOOS is appropriate for evaluating patients with patellar instability. The inability to detect changes in 15% and 46% of the patients in the *pain* and *ADL* subscales implies that a substantial number of patients score at the upper end of the scale *before* surgical treatment, leaving no room for improvement in these subscales. A possible reason for this might be lthe ow relevance of the items in these two subscales for patients with patellar instability.

The main concern about the ceiling effect, both pre‐ and postoperatively, is that the subscales are unable to distinguish between patients at the higher end of the scale. Making them clinically less meaningful for evaluation and follow‐up since the patient's clinical improvement cannot be quantified. It is well documented that patients with patellar instability have considerable problems with ADL and pain [1, 3, 10, 25, 29]. Therefore, the inability of the KOOS *pain* and *ADL* subscales to capture change/improvement demonstrated in this study suggests that the items in these subscales do not capture relevant complaints for these patients. If one compares pain‐related items in KOOS to the diagnose‐specific BPII score, some obvious differences are present. For example, both questionnaires probe about pain in relation to standing, however in the BPII, the question is connected to standing over a longer time > 30 minutes, while KOOS does not include duration. Another important aspect is that the problems patients with patellar instability face in ADL often are related to both psychological factors, that is, fear of new dislocations *and* physical factors.

The other subscales–*symptoms*, *sport/rec* and *QOL–*demonstrated some ability to detect short‐term improvement in knee function as no ceiling effects were observed in the preoperative scoring. However, the concerningly high levels of ceiling effect 6 months postoperatively displayed in the current implies that the KOOS is an inappropriate measurement instrument in long‐term evaluations in patients with patellar instability. This should not be surprising as floor and ceiling effects typically occur when an existing PROM is applied to a new population (e.g., patellar instability) that differs from the population for which the PROM was originally developed (in this case, patients with ACL injury) [6, p. 91, 24].

A floor effect was only seen in the preoperative KOOS *sport/rec* scoring. This makes sense given that the items in this subscale pertain to more knee‐demanding activities, such as squatting, running or twisting/pivoting, that patients perceive as exacerbating their patellar instability [26]. No such floor effect was seen in any of the subscales at 6 months after surgery.

Another concern about KOOS in a population of patients with patellar instability is that none of the 42 items directly address instability [24]. The major complaint of patients with this condition is the feeling/sensation of an unstable knee [10, 13, 26]. A PROM with the aim of evaluating knee function in patients with patellar instability should in our opinion include items that directly address instability, similar to the BPII or the NPI score.

The KOOS‐PF subscale might be a more appropriate choice for patients with patellar instability than the original KOOS. However, this version only concerns knee pain and comparable to the 42‐item version, none of the questions assesses instability.

The current suggested MICD values for the subscales *pain* and *QOL* are similar, but not identical, to previously reported values from comparable cohorts [23, 33]. In the subscales *symptoms, ADL* and *sport/rec*, the MICD values are inconsistent across the literature. Variations in the MICD in the *ADL* and *symptoms* subscale range from −5.15 to 10 and from 8.2 to 26.79, respectively. This discrepancy might be attributed to differences in follow‐up time—6 months in the current compared to 58 months in the study by Qaio et al. [23]—and the inclusion of only patients with MPFL‐R in the study by Walsh et al. [33].

There was a significant increase in all five subscales from pre‐ to 6 months postoperative. However, a statistically significant improvement may not reflect clinically relevant improvement or, more importantly, an improvement that patients perceive as significant. It is therefore essential to compare the increase in each subscale to the MICD values to determine if the change is meaningful to patients. In the current study, the amount of change exceeds the suggested MICD in all subscales except for KOOS *symptoms* and *sport/rec*. This indicates that the changes observed in the other subscales represent both, a statistically significant increase from pre‐ to 6 months postoperatively which is also meaningful to the patients.

This study has several limitations. First, calculating MICD using a distribution‐based approach is not the first‐line choice of method according to the COSMIN initiative [6, p. 246]. However, it provides an estimate to guide interpretation, as there are no suggested MICD values calculated with a visual anchor‐based MICD distribution method [6]. Future studies should verify the MICD with the recommended method. Second, the distribution between genders is skewed, with 74% of participants being female. While this may represent a limitation, it reflects the population experiencing this disorder, as females are more frequently affected by patellar instability. Third, in the current cohort, 16% of patients were under 16 years old. Knowing that KOOS originally was developed for young and middle‐aged patients, this could have affected the results. Ideally, would KOOS child [22] be a better alternative for the adolescent participants. We also included patients undergoing a spectrum of procedures, ranging from isolated MPFL‐R to trochleoplasty and tuberosity tibia osteotomy. Length of rehabilitation may differ between these procedures, and it should be recognised that this could have affected the KOOS scores at the 6 months follow‐up. Results from the current study should be interpreted with these factors in mind.

## CONCLUSION

The current study displayed a substantial ceiling effect in four out of five KOOS subscales acquired 6 months after surgery for patellar instability. Secondary to provide an MICD value for all subscales in patients treated for patellar instability with an individualised approach. The extensive ceiling effect is an important aspect to consider when deciding which PROMs to use in evaluations and follow‐ups after surgery for patellar instability. The findings in the current implies that the KOOS is not suited to evaluate the long‐term effect of treatment in patients with patellar instability.

## AUTHOR CONTRIBUTIONS

*Conception and design of the study, acquisition of data, analysis and interpretation of data and drafting of manuscript*: Trine Hysing‐Dahl. *Conception and design of the study, acquisition, analysis and critically revising manuscript*: Anne Gro Heyn Faleide. *Conception of study and critically revising manuscript*: Per Arne Skarstein Waaler. *Conception and design of the study and critically revising manuscript*: Eivind Inderhaug.

## CONFLICT OF INTEREST STATEMENT

The authors declare no conflicts of interest.

## ETHICS STATEMENT

The study was approved by the NSD (Norwegian Centre for Research Data) Data Protection Official for Research, Project Number 731409 and the Regional Committee for Medical and Health Research Ethics (ID: 2020/185067).

## Data Availability

Data are available on reasonable request. Quotations and further details are available from Trine Hysing‐Dahl at Trine. Hysing-Dahl@haraldsplass.no.
